# How the COVID-19 Pandemic Contributed to Diagnostic Bias

**DOI:** 10.7759/cureus.48282

**Published:** 2023-11-04

**Authors:** Antoine F AbdelMassih, Fatma el Zahraa M Gomaa, Rahaf Z AbuGhosh, Noura Shebl, Salah E Enab, Mohamed A ElBanna, Noha Ali

**Affiliations:** 1 Pediatric Cardiology, Cairo University, Cairo, EGY; 2 Pediatrics, Sheikh Khalifa Medical City, Abu Dhabi, ARE; 3 Pediatrics, Cairo University, Cairo, EGY; 4 Critical Care, Sheikh Khalifa Medical City, Abu Dhabi, ARE

**Keywords:** multi-system inflammatory disease in children (mis-c), cognitive bias, covid-19, coronary aneurysm, coronary av fistula

## Abstract

Diagnosis bias in the medical field is a recognized entity that can contribute to misdiagnoses and incorrect management. It remains a constant challenge that must be recognized and addressed. Several factors play a role in the formation of preconceptions which influence the physicians’ decision-making process. The aim of this paper is to present a case that was misdiagnosed and mistakenly managed due to diagnosis bias during the coronavirus disease 2019 (COVID-19) pandemic. We also suggest two ways to reduce the risk of diagnosis bias.

Multi-inflammatory syndrome of children (MIS-C) was described during the COVID-19 pandemic. The rise in the incidence of MIS-C masked the diagnosis of other diseases that present in a similar fashion. In this paper, we describe the case of a seven-year-old girl, who presented in 2020, with acute onset respiratory distress. Her chest images were suggestive of COVID-19 pneumonitis which prompted the physicians to complete the MIS-C workup by performing an echocardiogram. A large aneurysm of the left main artery was seen which led to a preliminary diagnosis of MIS-C. A repeat echocardiography, 48 hours after the initiation of MIS-C treatment, was suggestive of a large coronary fistula complicated by infective endocarditis and multiple septic pulmonary emboli. It can be inferred that the misdiagnosis occurred as a result of availability and premature-closure biases. Efforts to decrease such biases include group decision-making and using checklists during the assessment of a patient.

## Introduction

Coronary artery fistula is a rare congenital or acquired condition that involves abnormal communication between a coronary artery and either a cardiac chamber (coronary-cameral fistula) or any segment of the systemic or pulmonary circulations (coronary-arteriovenous fistula) [1]. Moreover, coronary dilatation can be a shared feature of other disorders, notably Kawasaki disease and the Kawasaki-like coronavirus disease 2019 (COVID-19)-induced multi-inflammatory syndrome of children (MIS-C) [2,3].

Clues to the diagnosis of coronary fistula include the presence of coronary dilatation as well as the detection of continuous flow by Doppler that connects the dilated coronary artery to the cardiac chamber [4]. 

On another note, diagnosis bias is one of the factors that plays a role in the initial impression and the subsequent management of a disease. It occurs when the diagnosis of a certain disease is misdirected due to prior diagnostic labels inflicted on the patient. These labels are influenced by multiple factors, such as the patient’s age and gender and other factors related to the region and era. Evidence suggests that tackling cognitive bias can drastically improve physicians' diagnostic abilities [5].

This report aims to illustrate a case of a delayed diagnosis of an infected coronary fistula due to a false diagnosis of MIS-C, which led to initial mismanagement.

## Case presentation

We describe the case of a seven-year-old female patient who presented in April 2020 with an acute onset of respiratory distress, associated productive cough, hemoptysis, and fever.

Her initial laboratory tests showed a marked elevation in the inflammatory markers, including a total leukocytic count of 22,000 x 10(9)/L and a C-reactive protein (CRP) of 161 mg/L. She had anemia (hemoglobin of 6.7 g/dL) with a normal reticulocyte count. Table 1 summarizes the available laboratory data on admission.

**Table 1:** Laboratory data of the patient on admission. CRP: C-reactive protein, ESR: erythrocyte sedimentation rate

	Patient’s values	Reference values
Hemoglobin	6.7 g/dL	10-15 g/dL
Total leucocytic count	22000/mm^3^	4,500 to 10,500/mm3
Neutrophils	15400/mm^3^	1000-8000/mm^3^
Lymphocytes	6600/mm^3^	1500-7000/mm^3^
Platelets	225000/mm^3^	150000-400000/mm^3^
CRP	161 mg/dL	<5 mg/dL
ESR 1^st^ hour	100 mm/hour	<15 mm/hour

**Figure 1:**
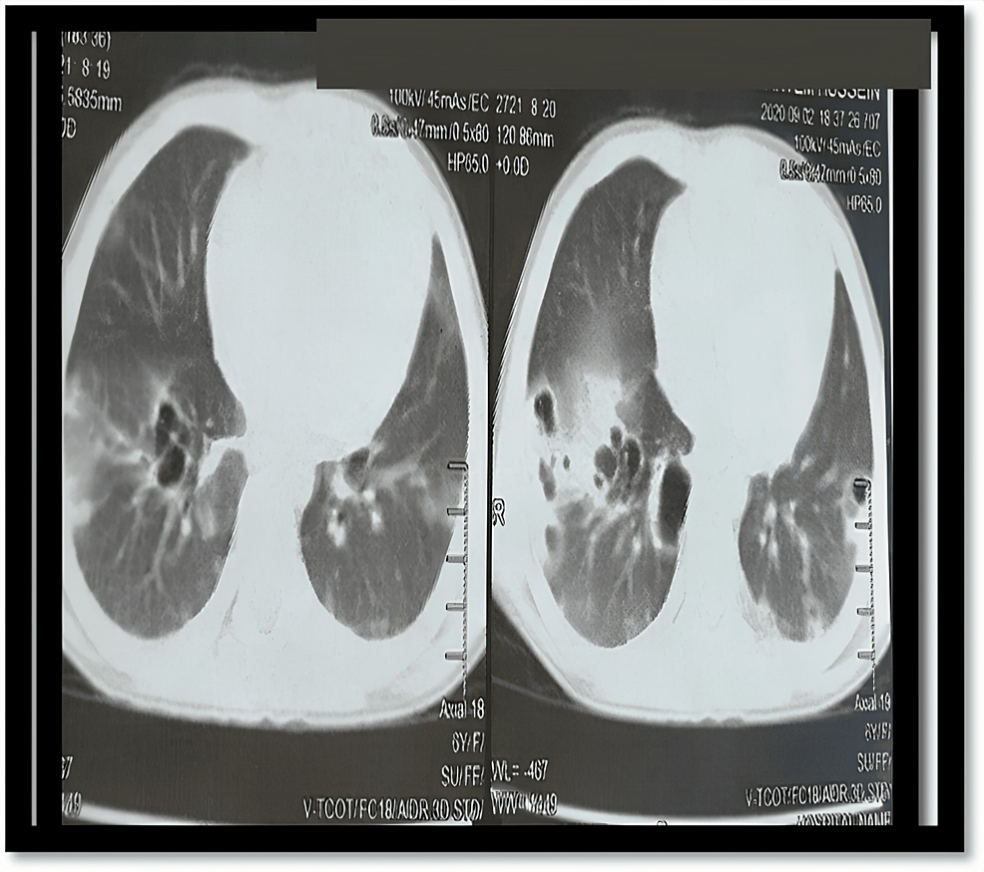
Axial computed tomography of the lungs of the patient on admission

Additionally, a CT scan of the chest was done, which showed bilateral scattered lung opacities mimicking COVID-19 pneumonitis (Figure 1).

Although COVID-19 pneumonitis was suspected, COVID-19 rapid antigen test and polymerase chain reaction (PCR) were not offered. That was secondary to the fact that diagnostic resources for COVID-19 testing in our country were, at that time, deficient due to the financial burden of the pandemic. As a consequence, the radiographic findings of the CT scan were relied upon for the impression of COVID-19 infection.

Owing to the fact that the patient presented during the COVID-19 pandemic, MIS-C was top in the differential list. Thus, an echocardiography was performed which showed a large aneurysm involving the left main coronary artery (LMCA). Video 1 shows the aneurysm involving the left main coronary artery.

**Video 1:** Coronary aneurysm of left main coronary artery (LMCA)

Therefore, there was an inclination towards the diagnosis of MIS-C, which led to the initiation of MIS-C management. A dose of intravenous immunoglobulins (IVIG) was given to the patient. 48 hours after the administration of IVIG, a repeat echocardiography was obtained which revealed a large filamentous tricuspid valve vegetation and a fistulous communication between the LMCA and the inferior surface of the right ventricle (RV) with a restrictive left to right shunt (continuous flow shunt). Video 2 shows the communication of the coronary fistula to the RV.

**Video 2:** Coronary fistulous communication to the right ventricle (RV)

**Figure 2:**
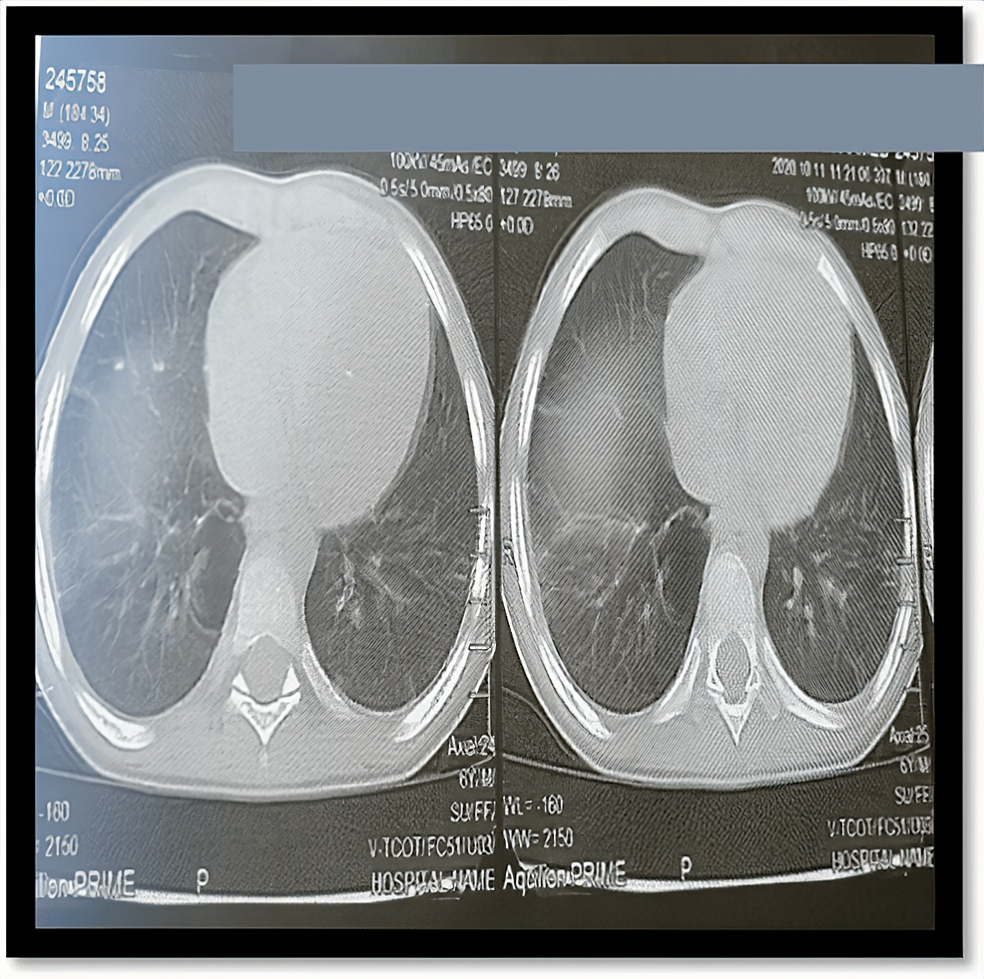
Axial computed tomography showing resolution of septic emboli seen in Figure 1.

The findings of the second echocardiography changed the diagnosis to a large coronary fistula complicated by infective endocarditis and multiple septic pulmonary emboli. Subsequently, the patient was appropriately treated with a 12-week period of the specific antibiotic regimen which led to dramatic improvements in the radiologic picture of the lung, in addition to the clinical symptoms of the patient. Video 3 shows the filamentous vegetations on the tricuspid valve before initiation of antibiotic therapy, Video 4 shows the gradual regression of vegetations after five weeks of antibiotic therapy, and Video 5 shows total resolution of vegetations on the tricuspid valve. Figure 2 also shows the complete resolution of the septic emboli from the lung field.

**Video 3:** Filamentous vegetations on the tricuspid valve (TV) in infected coronary fistula

**Video 4:** Gradual regression of vegetations on the tricuspid valve (TV) after six weeks of antibiotic therapy in infected coronary fistula

**Video 5:** Total resolution of vegetations on the tricuspid valve (TV) after 12 weeks of antibiotic therapy in infected coronary fistula

## Discussion

Multiple cognitive biases, such as confirmation and anchoring biases, are well known to affect a physician’s ability to gather evidence and shape their decision-making process. In fact, more than 100 different biases have been identified to play a role in the practice of medicine. It has been estimated that the rate of misdiagnosis in medicine secondary to biases is around 10-15% [6].

When correlating this to the case presented in this study, one can easily recognize two types of biases: the availability bias and the premature closure bias. Furthermore, availability bias happens when a certain diagnosis is selected due to frequency or recency, whereas premature closure occurs when a diagnosis and an intervention are made early or “prematurely” without going thoroughly through the whole differential diagnosis.

Applying this concept to this case, it can be concluded that availability bias played a role in making a diagnosis of MIS-C as the patient presented during the COVID-19 pandemic, where the surge of MIS-C cases made the diagnosis more prevalent. Coronary dilatation with signs of hyper-inflammation was considered pathognomonic for MIS-C until proven otherwise.

It can also be noted that there was a tendency towards premature closure without a thorough, systematic assessment of all possibilities. A careful study of the CT scan images would prove that the changes noted are not characteristic of COVID-19 pneumonitis but rather point towards septic emboli. Additionally, the initial echocardiography might not have included all views due to its “premature closure” after finding a coronary dilatation, which is suggestive of MIS-C.

The term “bias” is often thought to have a negative connotation when used in the medical field. Nevertheless, it is crucial to understand that preconceptions are developed by the human brain to speed up daily processes. The unconscious thought processes enable one to recognize common, repeated patterns, which in turn leaves more effort for conscious thought processing when dealing with novel challenges [7].

In efforts to limit diagnostic bias, certain strategies can be implemented. These include getting a second opinion or group decision-making and using checklists during clinical, laboratory, or imaging assessments of a patient. For instance, in the case presented, repetition of the echocardiography by another operator after two days helped reveal the diagnosis. In addition to that, setting a checklist of the possible causes of coronary dilatation could have prevented the misdiagnosis that took place initially [8-10].

## Conclusions

It is important to remember that “not all that happened during the pandemic is necessarily due to the pandemic”. Detailed history-taking and full systematic physical examinations are the mainstays of any diagnosis. Physicians should always perform their history and full physical examination on their patients to avoid getting affected by any previous diagnosis or any trend to prevent “diagnostic bias”.
